# Interaction of Arabidopsis Trihelix-Domain Transcription Factors VFP3 and VFP5 with Agrobacterium Virulence Protein VirF

**DOI:** 10.1371/journal.pone.0142128

**Published:** 2015-11-16

**Authors:** Elena García-Cano, Shimpei Magori, Qi Sun, Zehong Ding, Sondra G. Lazarowitz, Vitaly Citovsky

**Affiliations:** 1 Department of Biochemistry and Cell Biology, State University of New York, Stony Brook, New York, United States of America; 2 Computational Biology Service Unit, Cornell University, Ithaca, New York, United States of America; 3 Department of Plant Pathology and Plant-Microbe Biology, Cornell University, Ithaca, New York, United States of America; Indiana University, UNITED STATES

## Abstract

Agrobacterium is a natural genetic engineer of plants that exports several virulence proteins into host cells in order to take advantage of the cell machinery to facilitate transformation and support bacterial growth. One of these effectors is the F-box protein VirF, which presumably uses the host ubiquitin/proteasome system (UPS) to uncoat the packaging proteins from the invading bacterial T-DNA. By analogy to several other bacterial effectors, VirF most likely has several functions in the host cell and, therefore, several interacting partners among host proteins. Here we identify one such interactor, an Arabidopsis trihelix-domain transcription factor VFP3, and further show that its very close homolog VFP5 also interacted with VirF. Interestingly, interactions of VirF with either VFP3 or VFP5 did not activate the host UPS, suggesting that VirF might play other UPS-independent roles in bacterial infection. To better understand the potential scope of VFP3 function, we used RNAi to reduce expression of the *VFP3* gene. Transcriptome profiling of these *VFP3*-silenced plants using high-throughput cDNA sequencing (RNA-seq) revealed that VFP3 substantially affected plant gene expression; specifically, 1,118 genes representing approximately 5% of all expressed genes were significantly either up- or down-regulated in the *VFP3* RNAi line compared to wild-type Col-0 plants. Among the 507 up-regulated genes were genes implicated in the regulation of transcription, protein degradation, calcium signaling, and hormone metabolism, whereas the 611 down-regulated genes included those involved in redox regulation, light reactions of photosynthesis, and metabolism of lipids, amino acids, and cell wall. Overall, this pattern of changes in gene expression is characteristic of plants under stress. Thus, VFP3 likely plays an important role in controlling plant homeostasis.

## Introduction

Agrobacterium genetically modifies plants in nature to cause crown gall disease [1]. Under laboratory conditions, Agrobacterium also can transform practically any eukaryotic species, from fungal to human cells [2]. To initiate infection, Agrobacterium exports single-stranded molecules of the transferred DNA (T-DNA) into its target cells, as well as several types of virulence (Vir) protein effectors that actively participate in the transformation process [3, 4]. One such exported bacterial effector is VirF [5], which is an F-box protein [6]. Studies suggest that VirF recognizes and induces degradation by the ubiquitin/proteasome system (UPS) of the plant protein VIP1 and its associated bacterial effector VirE2 [7, 8], which likely package the T-DNA into a transfer (T) complex for nuclear import and chromatin targeting [9–11]. Thus, one function of VirF may be to uncoat these associated proteins from the T-DNA molecule via the SCF^VirF^ pathway prior to integration of the T-DNA into the host cell genome [7, 8]. Interestingly, because VirF is a host range determinant, it is not essential for infection of some plant species [12, 13], which presumably encode their own F-box proteins [14] that can fulfill this function, such as VBF in Arabidopsis [14].

Many bacterial effectors are multifunctional proteins [15–18]. Thus, it is likely that VirF has other functions in the host cell. To gain insight into these potential additional functions, we performed a systematic search for VirF-interacting proteins in Arabidopsis. Here, we describe two such interactors, VFP3 and its close homolog VFP5, which are members of the trihelix-domain family of transcription factors. VFP3/VFP5 interactions with VirF were confirmed *in planta*, and the effects of RNAi silencing of *VFP3* on the Arabidopsis transcriptome were examined.

## Results Identification of VFP3

**Fig 1:**
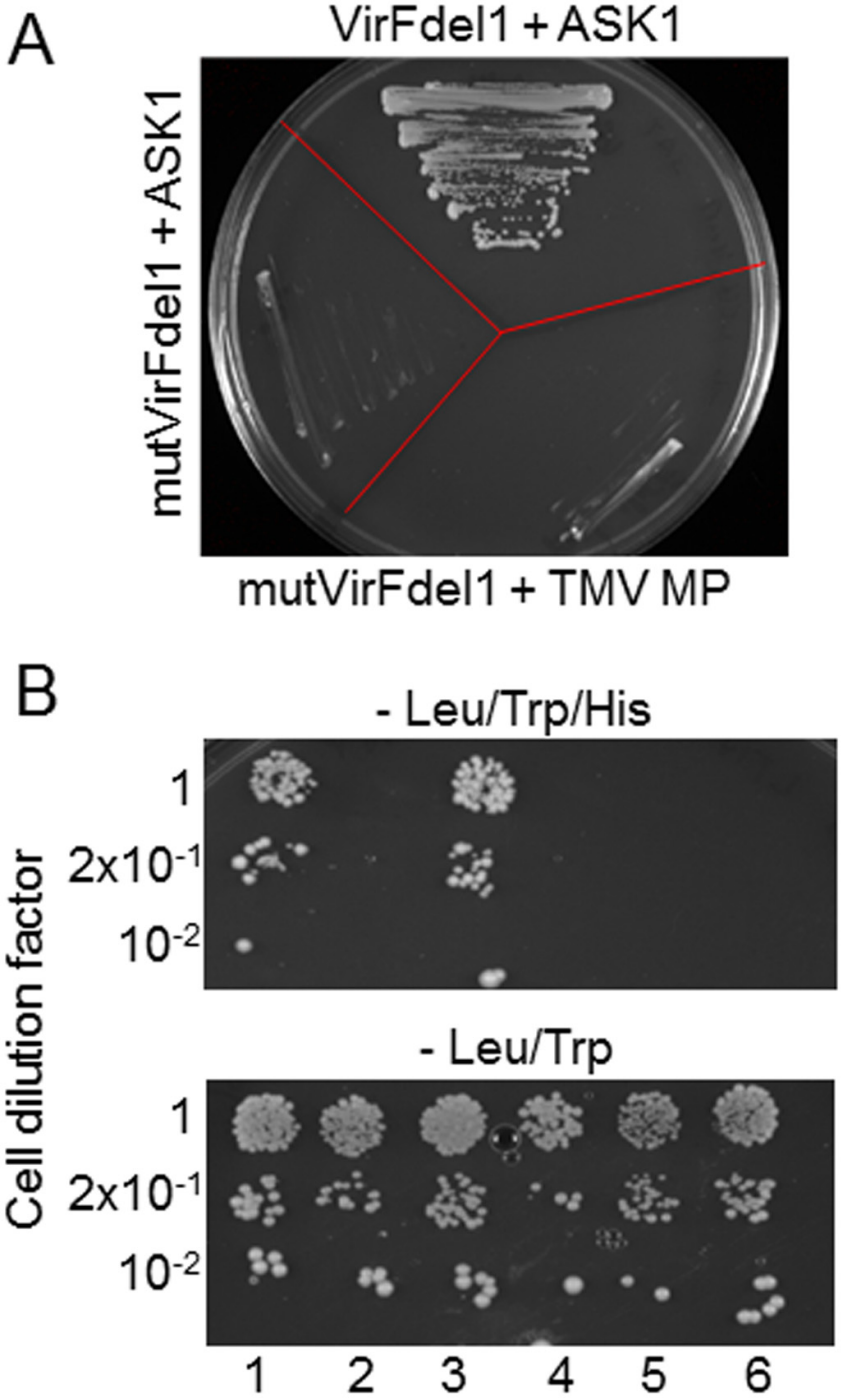
Specific interaction between VFP3 and VirFdel1 in the yeast two-hybrid system. (A) Characterization of the VirF-based bait, mutVirFdel1. Yeast cells were grown in the absence of leucine, tryptophan, and histidine. (B) VirFdel1-VFP3 interaction. The indicated dilutions of yeast cells were grown in the absence of histidine, tryptophan, and leucine (top) or in the absence of tryptophan and leucine (bottom). Lane 1, LexA-mutVirFdel1 + Gal4AD-VFP3; lane 2, LexA-mutVirFdel1 + Gal4AD-TMV MP; lane 3, LexA-VirFdel1 + Gal4AD-VFP3; lane 4, LexA-VirFdel1 + Gal4AD-TMV MP; lane 5, LexA-AtCUL1 + Gal4AD-VFP3; Lane 6, LexA-AtCUL1 + Gal4AD-TMV MP. Growth in histidine-deficient medium represents selective conditions for protein-protein interaction.

To identify VirF-interacting proteins, we used a mutated form of VirF, designated mutVirFdel1, as bait in a yeast two-hybrid screen of our Arabidopsis cDNA library [19–21]. Since VirF is an F-box protein, its major partners in plant cells include members of the Skp1/ASK family that associate with F-box proteins in SCF complexes [6, 7]. Because we aimed to search for novel VirF interactors, we introduced two point mutations into the VirF F-box domain [6] to generate the mutant mutVirF, which is unable to interact with ASK proteins[6]. In addition, we deleted the 15 N-terminal residues of VirF so that the bait would not self activate. Fig 1A shows that the resulting bait, mutVirFdel1, indeed did not self-activate when coexpressed with the unrelated movement protein of *Tobacco mosaic virus* (TMV MP), and it did not interact with Arabidopsis ASK1, whereas VirFdel1 with its intact F-box motif did interact with ASK1.

Using mutVirFdel1, we screened 3.97 x 10^6^ transformants and isolated three different cDNA clones encoding potential VirF-interacting proteins (VFPs). Here, we focus on one clone designated VFP3, which was isolated in two independent screening experiments. Fig 1B shows that VFP3 interacted with VirF in the yeast two-hybrid assay, and that this interaction was independent of the VirF F-box domain as it occurred both with VirFdel1 and with mutVirFdel1. Furthermore, this interaction was specific as it was not observed with the unrelated control proteins TMV MP and AtCUL1 (Fig 1B).

**Fig 2:**
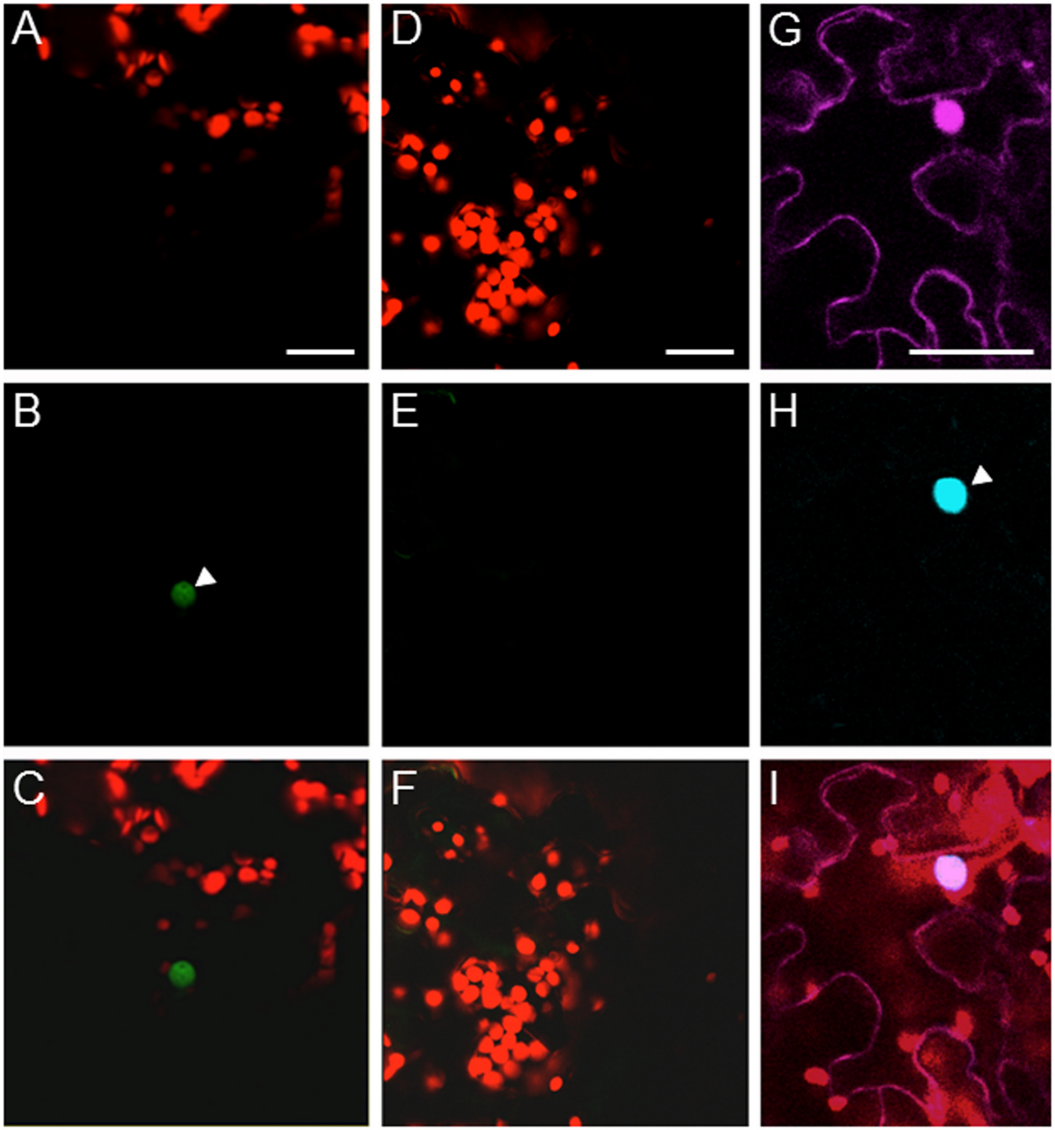
VFP3 interacts with VirF in the cell nucleus. (A-C) BiFC assay for the VFP3-VirF interaction in planta. Constructs encoding nYFP-VFP3 and cYFP-VirF were coexpressed in microbombarded *N*. *benthamiana* leaves. (A) Plastid autofluorescence. (B) YFP signal. (C) Merged plastid autofluorescence and YFP signals. (D-F) BiFC assay for the VFP3-VBF interaction in planta. Constructs encoding nYFP-VFP3 and cYFP-VBF were coexpressed in microbombarded *N*. *benthamiana* leaves. (D) Plastid autofluorescence. (E) YFP signal. (F) Merged plastid autofluorescence and YFP signals. (G-I) Subcellular localization of CFP-tagged VFP3 coexpressed with free DsRed2 in agroinfiltrated *N*. *benthamiana* leaves. (G) DsRed2 signal. (H) CFP signal. (I) Merged plastid autofluorescence, DsRed2 and CFP signals. Location of the cell nucleus is indicated by a white arrowhead. All images are projections of single confocal sections. Scale bars, 20 μm.

We next used bimolecular fluorescence complementation (BiFC) to detect VirF-VFP3 interaction and the subcellular localization of the VirF-VFP3 complexes within plant cells [22, 23]. VirF and VFP3 were tagged with C-terminal and N-terminal fragments of YFP, respectively, and transiently coexpressed in *N*. *benthamiana* leaves. Fig 2A–2C shows that cYFP-VirF and nYFP-VFP3 interacted in plant cells, producing the BiFC signal. This interaction predominantly occurred in the cell nucleus. As expected, co-expression of cYFP-VirF with nYFP-TMV MP and of nYFP-VFP3 with cYFP-AtCUL1 failed to reconstitute the BiFC fluorescence (data not shown). Because the potential VirF function in proteasomal uncoating of the T-DNA can be mimicked by the Arabidopsis F-box protein VBF [8, 14], we also used BiFC to examine whether VBF can interact with VFP3; however, this interaction was not observed under our experimental conditions (Fig 2D–2F). Thus, the interaction with VFP3 most likely is specific for bacterial VirF.

We also determined the subcellular localization of VFP3 in plant cells. To this end, VFP3 was tagged with CFP and transiently coexpressed with free DsRed2. Fig 2G shows that free DsRed2 partitioned between the cell cytoplasm and the nucleus, conveniently identifying both of these cellular compartments. CFP-VFP3 was observed exclusively in the nucleus (Fig 2H) colocalizing with nuclear DsRed2 (Fig 2I).

### VFP3 belongs to the plant-specific family of trihelix-domain transcription factors

**Fig 3:**
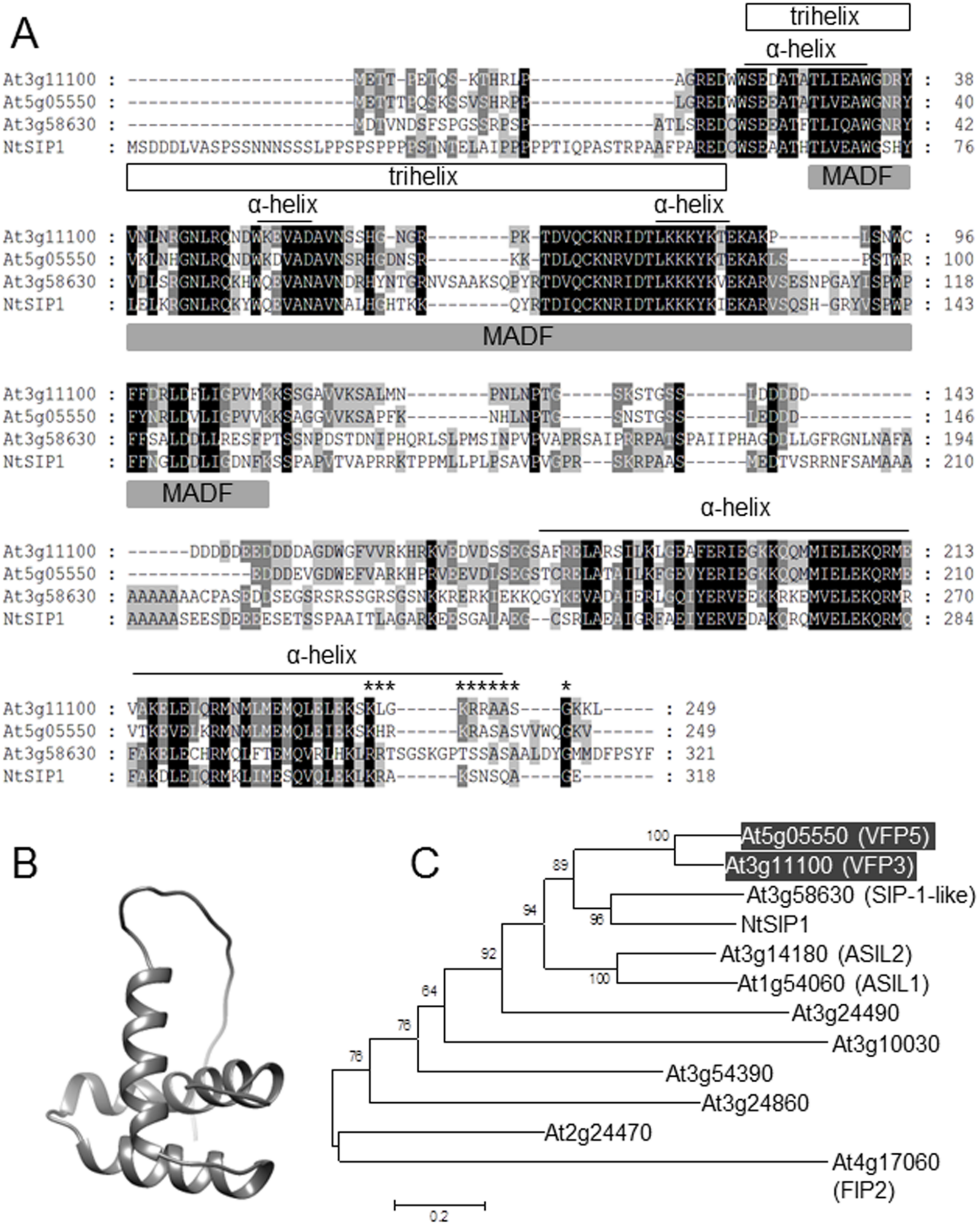
Amino acid sequence analysis of VFP3. (A) Sequence alignment of VFP3 and its homologs from Arabidopsis. The deduced amino acid sequence of VFP3 (At3g11100) was aligned with the sequences of proteins encoded by At5g05550 (VFP5) and At3g58630 of Arabidopsis and of tobacco NtSIP1 (GenBank accession number BAB83610.1) using ClustalX (ver. 2.1) (http://www.clustal.org/clustal2/). Three α-helices of the trihelix domain, delineated with an open box, and the fourth C-terminal α-helical region were predicted using the Garnier-Robson-Osguthorpe (GOR) algorithm [26]. The MADF domain, predicted by InterPro (http://www.ebi.ac.uk/interpro), is delineated with a gray box. Asterisks indicate the putative monopartite NLS predicted by cNLS Mapper (nls-mapper.iab.keio.ac.jp). Identical residues in the aligned sequences are highlighted in white letters on black/dark gray background and similar residues are shaded in gray. (B) Ribbon diagram of the trihelix domain VFP3 showing the three predicted helical structures was constructed using the Hhpred (http://toolkit.tuebingen.mpg.de/) [59] and UCSF Chimera tools [60]. (C) Phylogenetic tree of the members of the SIP1 clade of Arabidopsis trihelix transcription factors and tobacco NtSIP1. VFP3 (At3g11100) and its close homolog VFP5 (At5g05550) are highlighted by a shaded box and white letters. Known gene names are indicated in parenthesis next to their locus names. The evolutionary history was inferred using the Neighbor-Joining method [61]. The optimal tree with the sum of branch length of 6.60887794 is shown. The percentage of replicate trees in which the associated taxa clustered together in the bootstrap test (1,000 replicates) are shown next to the branches [62]. The tree is drawn to scale, with branch lengths in the same units as those of the evolutionary distances used to infer the phylogenetic tree. The evolutionary distances were computed using the Poisson correction method [63] and are in the units of the number of amino acid substitutions per site. All positions containing gaps and missing data were eliminated. There were a total of 199 positions in the final dataset. Evolutionary analyses were conducted using the Molecular Evolutionary Genetics Analysis tool (MEGA, version 6.0.5 for Mac OS) (http://www.megasoftware.net) [64], which also generated this description of the analysis. Bar, 0.2 amino acid substitutions per site.

Sequence analysis of the full-length VFP3 cDNA predicted a single open reading frame (ORF) encoding a protein of 249 amino acid residues with a relative molecular mass of 27.3 kDa. The *VFP3* gene (At3g11100) is annotated as a member of the trihelix gene family of transcription factors in the database of Arabidopsis transcription factors (DAFT) (http://datf.cbi.pku.edu.cn) [24] as well as in the recent comprehensive description of this family [25]. Prediction of secondary structure suggested that amino acid residues 24–41, 48–62, and 70–88 of VFP3 form three α-helices with short intervening loops, i.e., a trihelix domain, and the region between amino acid residues 182 and 291 contains another α-helix (Fig 3A); this latter C-terminal helical domain is found in most trihelix proteins and is likely involved in their multimerization [25]. Predictions of secondary structure using the Garnier-Robson-Osguthorpe (GOR) algorithm [26] suggested that residues 22 to 90 of VFP3 form three α-helices with short intervening loops (Fig 3A). The trihelix domain was also evident in the predicted three-dimensional structure of this VFP3 region (Fig 3B).

Consistent with its putative function as a transcription factor and its nuclear localization in plant cells (see Fig 2), VFP3 contains a predicted a monopartite nuclear localization signal (NLS) in its C-terminal region between positions 237 and 246 (Fig 3A). Finally, the VFP3 region between amino acid residues 29 to 110, contains a predicted MADF (myb/SANT-like domain in Adf-1) domain (Fig 3A), a module that directs sequence-specific binding of this family of transcription factors to their DNA target sites, which comprise multiple trinucleotide repeats (http://www.ebi.ac.uk/interpro/entry/IPR006578;jsessionid=12C4F0FAB54A32F4D9ABF237F102CF4F).

Trihelix-domain transcription factors are plant-specific proteins, thought to have evolved after the divergence of plants and animals [27]. There are 30 members of this family encoded by Arabidopsis, and these are divided into five clades [25, 28]. VFP3 belongs to the SIP1 clade, which comprises 11 proteins [25], or to clade V according to another classification [28]. A phylogenetic tree constructed using these protein sequences and their tobacco homolog NtSIP1, which was previously implicated in Agrobacterium infection [29, 30], revealed several distinct subclades, with the Arabidopsis proteins encoded by At5g05550 and At3g58630, and the tobacco NtSIP1, being most closely related to VFP3 (Fig 3A and 3C). Specifically, the protein products of At5g05550 and At3g58630, and NtSIP1 exhibited 71.9%, 34.5%, and 35.7% identity, respectively, to VFP3. Those regions showing identity included most of the trihelix domain and part of the putative NLS residues (Fig 3A).

### VirF interacts with the VFP3 homolog VFP5 encoded by *At5g05550*

**Fig 4:**
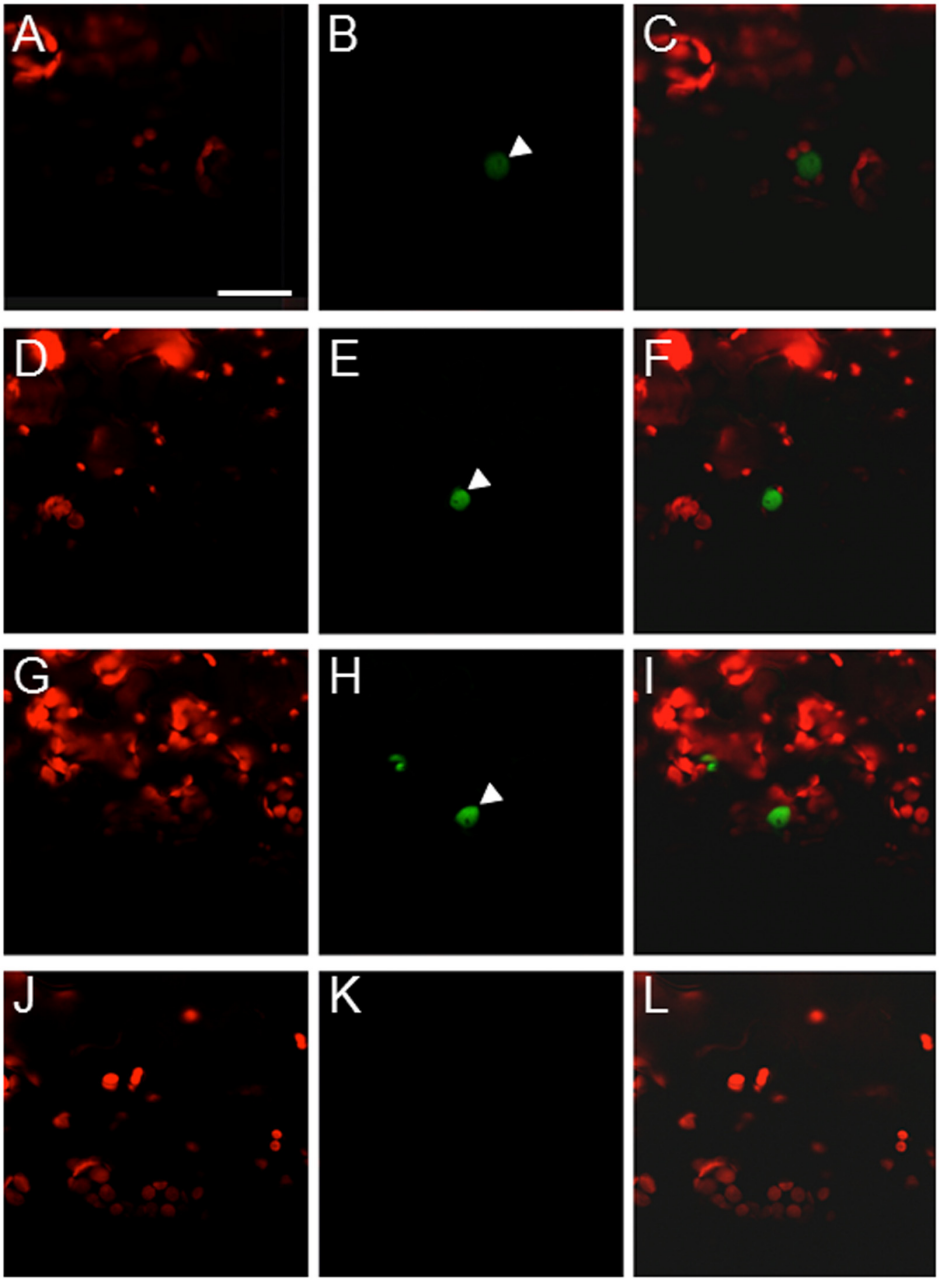
VirF and 6b interact with the VFP3 homolog, VFP5, in planta. (A-C) BiFC assay for the cYFP-VirF interaction with nYFP-VFP5. (D-F) BiFC assay for the cYFP-6b interaction with nYFP-VFP3. (G-I) BiFC assay for the cYFP-6b interaction with nYFP-VFP5. (J-L) BiFC assay for the cYFP-6b interaction with nYFP-At3g58630. Constructs encoding the tested proteins were coexpressed in microbombarded *N*. *benthamiana* leaves. (A, D, G, J) Plastid autofluorescence. (B, E, H, K) YFP signal. (C, F, I, L) Merged plastid autofluorescence and YFP signals. Location of the cell nucleus is indicated by a white arrowhead. All images are projections of single confocal sections. Scale bars, 20 μm.

That VFP3 is closely related to the protein predicted to be encoded by the Arabidopsis *At5g05550* gene suggested that this protein might also interact with VirF. We tested this idea using BiFC to detect interaction between cYFP-tagged VirF and the nYFP-tagged protein product of the *At5g05550* gene in *N*. *benthamiana* leaf cells. Fig 4A–4C shows that, indeed, VirF interacted with the *At5g05550*-encoded protein in plant cells and that the interacting complexes accumulated in the cell nucleus, similar to VirF-VFP3 interaction (see Fig 2). Thus, we designated the *At5g05550* gene product VFP5. In addition, the tobacco homolog of VFP3, NtSIP1, has been shown to interact with the 6b oncogene protein encoded by Agrobacterium T-DNA [29, 30]. We therefore examined whether 6b could also interact with VFP3 and VFP5. Fig 4 demonstrates that 6b interacted with both VFP3 (panels D-F) and VFP5 (panels G-I). These interactions were specific because they were not observed with nYFP-tagged protein product of *At3g58630* (Fig 4J–4L), an Arabidopsis trihelix protein more evolutionarily distant to VFP3 than VFP5 (see Fig 3C).

### VirF does not destabilize VFP3 or VFP5

**Fig 5:**
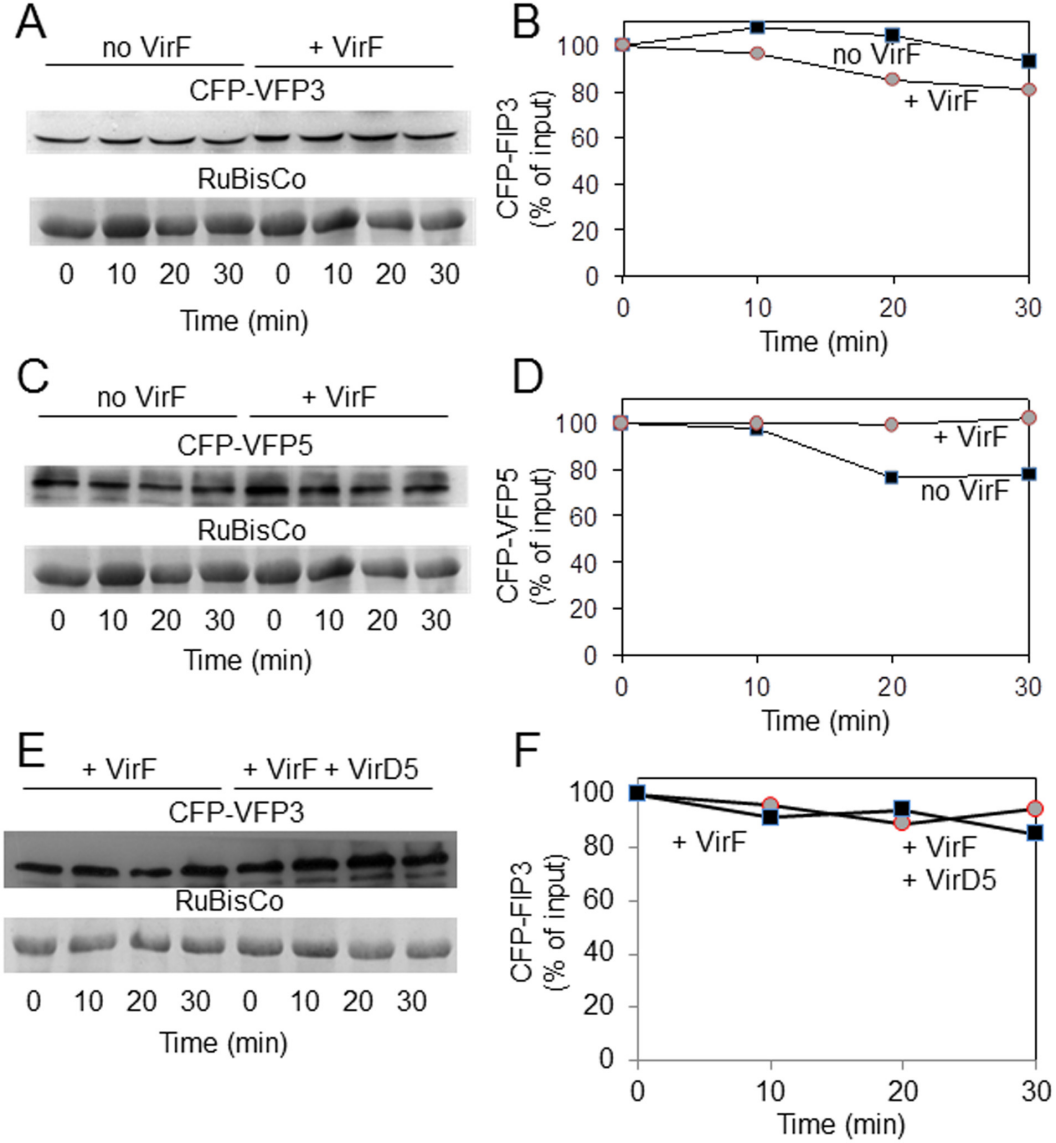
VirF does not destabilize VFP3 or VFP5 in a cell-free degradation assay. (A) Western blot analysis of CFP-VFP3 following coexpression with Myc-VirF. (B) Quantification of CFP-VFP3 accumulation described in (A). (C) Western blot analysis of CFP-VFP5 following coexpression with Myc-VirF. (D) Quantification of CFP-VFP5 accumulation described in (C). (E) Western blot analysis of CFP-VFP3 following coexpression with Myc-VirF and free VirD5. (F) Quantification of CFP-VFP3 accumulation described in (E). The tested proteins were coexpressed in *N*. *benthamiana* leaves, cell extracts were prepared and incubated for the indicated periods of time. CFP-VFP3 and CFP-VFP5 were detected by anti-CFP antibody, and RuBisCo was detected by Coomassie blue staining. The putative RuBisCo large chain was used as loading control and as reference for normalization of relative protein amounts. Each experiment was performed at least twice with similar results.

VirF is an F-box protein that promotes proteasomal destabilization of at least one of its host cell interactors, VIP1 [7, 8]. We therefore examined whether VirF would destabilize VFP3 and/or VFP5, with which it also interacts. To this end, we analyzed the stability of VFP3 or VFP5 in the presence or absence of VirF using our cell-free degradation assay [31]. Cell extracts were prepared from *N*. *benthamiana* plants that transiently expressed VFP3 or VFP5, each tagged with CFP, in the presence or absence of Myc-tagged VirF, and the levels of CFP-VFP3 or CFP-VFP5 were analyzed by western blotting. It is worth noting that, in our experience [7, 14, 32], neither epitope nor GFP-based tags interfere with VirF/VBF-mediated proteasomal degradation. Fig 5 shows that VFP3 protein accumulated to comparable levels in extracts from plants that expressed CFP-VFP3 alone or together with Myc-VirF (panels A, B). Similarly, Myc-VirF did not affect the levels of accumulation of VFP5: comparable levels of this protein accumulated in the absence and presence of VirF (Fig 5C and 5D). Note that very minor bands observed in some lanes may due to low levels of antibody cross-reactivity with some of the plant proteins.

We then examined whether another bacterial effector, VirD5, which has been shown to bind to and stabilize VirF [32], might cooperate with VirF to destabilize VFP3. Fig 5E and 5F shows that coexpression of free VirD5 with Myc-VirF and CFP-VFP3 did not affect the accumulation of CFP-VFP3. Collectively, these results suggest that VirF binding to VFP3 and VFP5 most likely does not act to destabilize these transcription factors.

### RNAi silencing of *VFP3* expression in Arabidopsis does not affect Agrobacterium tumorigenicity

**Fig 6:**
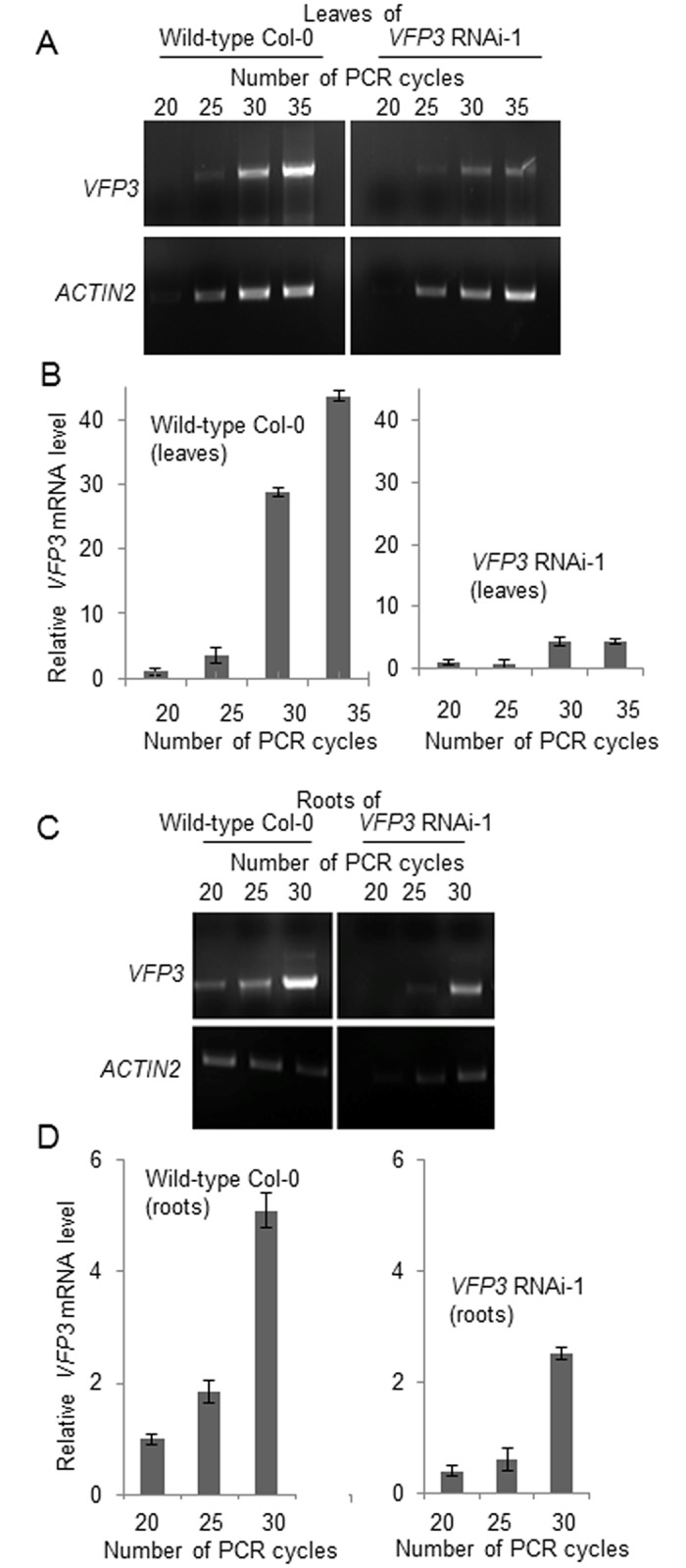
Reduction of *VFP3* gene expression in *VFP3* RNAi-1 plants. (A) Semi-quantitative RT-PCR analysis of the *VFP3* transcript levels in leaves of the wild-type Col-0 and *VFP3* RNAi-1 plants. *ACTIN2* was used as internal reference. (B) Quantification of *VFP3* transcript levels described in (A) normalized to the levels of the *ACTIN2* reference. (C) Semi-quantitative RT-PCR analysis of the *VFP3* transcript in roots of the wild-type Col-0 and *VFP3* RNAi-1 plants. *ACTIN2* was used as internal reference. (D) Quantification of *VFP3* transcript levels described in (C) normalized to the levels of the *ACTIN2* reference. The data represent average values of three independent experiments with indicated standard deviations.

To better understand the role of *VFP3* in Agrobacterium infection in particular and in plant physiology in general using reverse genetics. Because T-DNA insertion mutants in the *VFP3* gene are not presently available, we used RNAi to suppress endogenous expression of the *VFP3* gene in Arabidopsis Col-0. We identified five independent RNAi-silenced *VFP3* knockdown lines, which developed comparable levels of suppression (data not shown), and analyzed one of these lines, designated *VFP3* RNAi-1, in detail. Fig 6 shows semi-quantitative RT-PCR analysis of *VFP3* expression in leaf and root extracts from *VFP3* RNAi-1 plants as compared to wild-type Col-0 plants. *VFP3* transcript levels were relatively high in wild-type Col-0 leaf tissue whereas *VFP3* expression was suppressed 10-fold in *VFP3* RNAi-1 leaves (Fig 6A and 6B). In control experiments, transcripts of the constitutively expressed *ACTIN2* gene accumulated to comparable levels in wild-type Col-0 and *VFP3* RNAi-1 leaves (Fig 6A). In roots, *VFP3* transcript levels were reduced approximately two-fold in *VFP3* RNAi-1 compared to wild-type Col-0 plants (Fig 6C and 6D). Thus, RNA silencing of *VFP3* expression occurred both in leaves and roots, albeit with different efficiencies. We also could not detect any overt changes in morphology or development of the *VFP3* RNAi-1 plants (data not shown); potentially, such phenotypes might develop only under specific conditions, such as abiotic or biotic stress.

**Fig 7:**
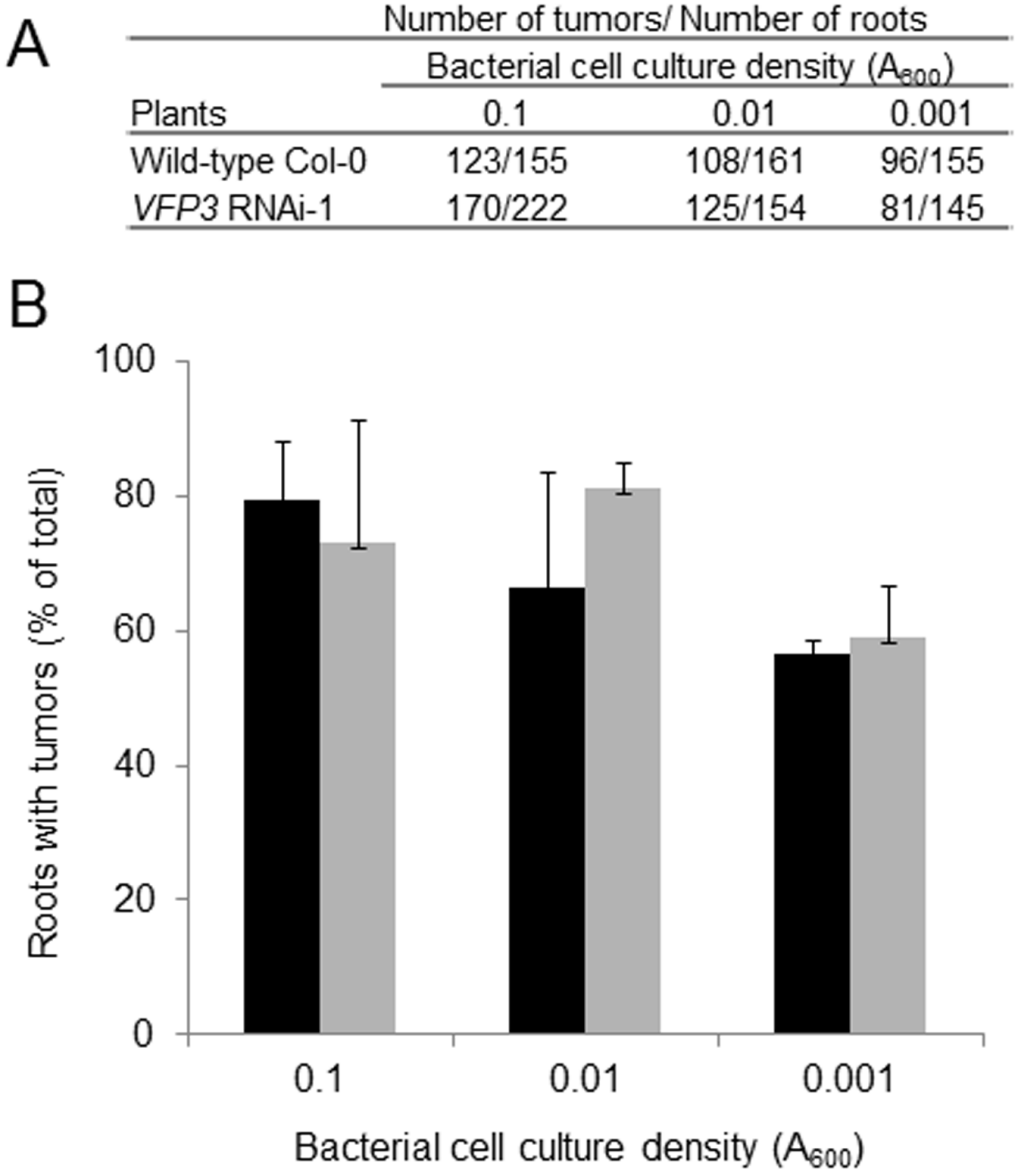
Suppression of *VFP3* gene expression in *VFP3* RNAi-1 plants has no detectable effect on their genetic transformation by Agrobacterium. Root explants were infected with Agrobacterium cultures at the indicated optical densities. (A) Numbers of tumors and roots scored for each plant. (B) Tumorigenicity expressed as percent of roots showing tumors. Black bars, wild-type plants; gray bars, *VFP3* RNAi-1 plants. Standard deviations are indicated.

Next, we used the *VFP3* RNAi-1 line to examine the effect of suppressing *VFP3* expression on susceptibility to Agrobacterium-mediated genetic transformation. To assess transformation, we utilized the root tumor assay [33] with standard (A_600_ = 0.1) to low (A_600_ = 0.01–0.001) densities of bacterial cell cultures to detect potential differences at subsaturating transformation efficiencies. Both the wild-type and *VFP3* RNAi-1 plants were susceptible to Agrobacterium tumorigenicity to similar extent at all inoculation densities, developing comparable numbers of tumors (Fig 7A) on 60–80% of all inoculated roots (Fig 7B). Thus, under our experimental conditions, suppressing *VFP3* expression, at least at the relatively low levels observed in roots (see Fig 6), did not have a significant effect on the ability of Agrobacterium to elicit tumors.

### Effect of suppressing *VFP3* expression on the Arabidopsis transcriptome

We used RNA-seq analyses to assess the global effects of suppressing *VFP3* expression on the transcription profile of Col-0 plants. We used the same RNA samples from leaves that were characterized (Fig 6A and 6B) and analyzed the sequencing results based on MapMan annotations. In modern plant science, there are two widely used ontology techniques: Gene Ontology (GO) and MapMan. GO was developed as a species-nonspecific approach, whereas MapMan was purposefully designed to analyze plant-specific pathways and processes. Thus, MapMan has been used to analyze transcription profiles in diverse plant species such as maize [34], tomato [35], potato [36] and, more recently, in Arabidopsis [37].

**Fig 8:**
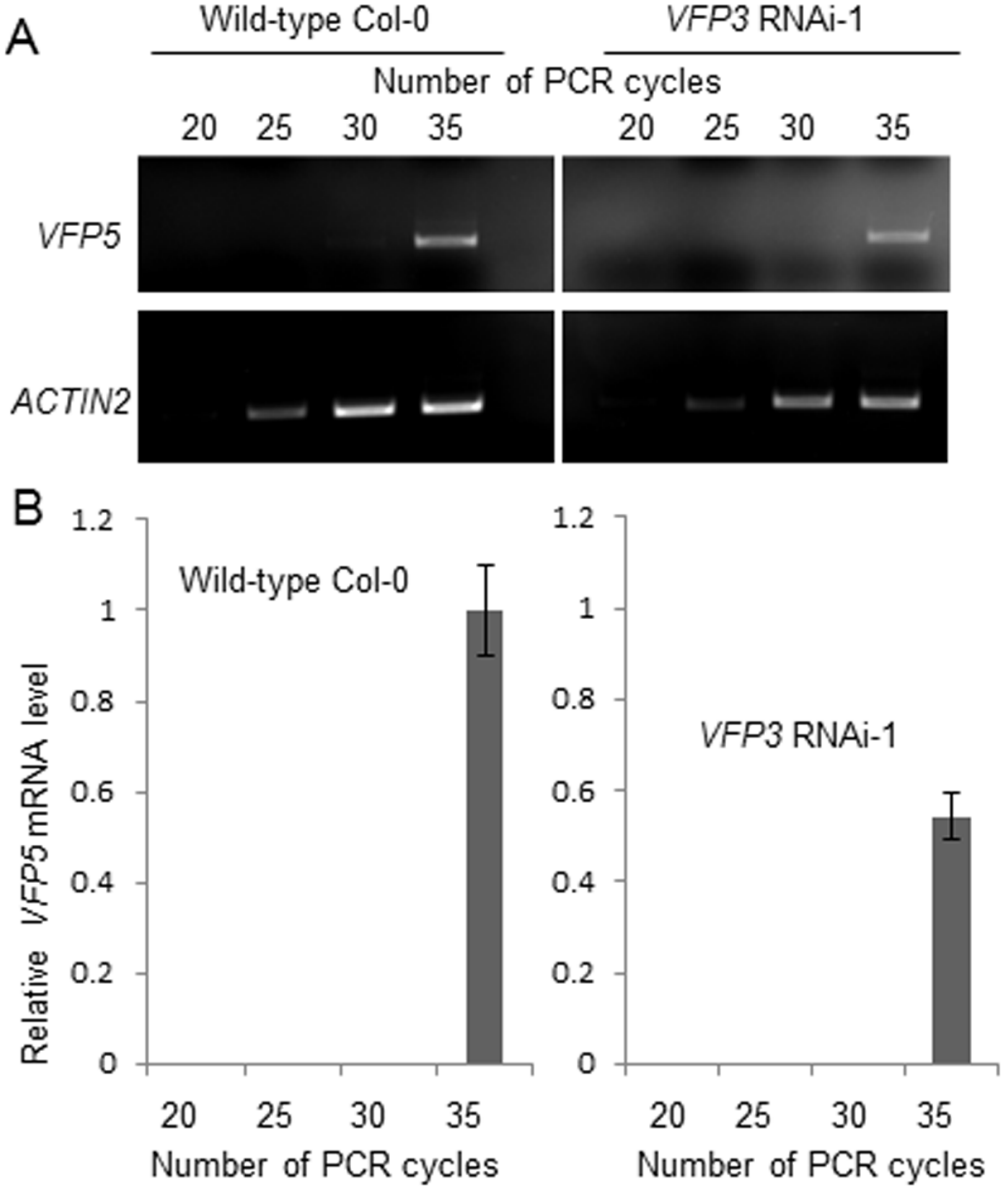
Reduction of *VFP5* gene expression in *VFP3* RNAi-1 plants. (A) Semi-quantitative RT-PCR analysis of the *VFP5* transcript in leaves of the wild-type Col-0 and *VFP3* RNAi-1 plants. *ACTIN2* was used as internal reference. (B) Quantification of *VFP5* transcript levels described in (A) normalized to the levels of the *ACTIN2* reference. The data represent average values of three independent experiments with indicated standard deviations.

Using DESeq [38] to calculate differentially expressed genes (DEGs), we identified statistically significant differences in gene expression between the two lines. Specifically, we identified a total of 1,118 genes that were either up- or down-regulated (FDR < 0.001 and log_2_FC > 2) in *VFP3* RNAi-1 plants compared to wild-type Col-0 plants, representing ~5% of the 22,270 expressed genes with mapped reads >5 in at least one sample. These transcriptome changes most likely also reflect at least some of the effects of the *VFP5* gene, which exhibits a high degree of sequence identity to *VFP3* (see Fig 3A), and thus can be silenced in the *VFP3* RNAi-1 line. Indeed, RT-PCR of leaf tissue extracts from *VFP3* RNAi-1 plants demonstrated a two-fold decrease in the levels of the *VFP5* transcript (Fig 8). Note that we did not include *VFP3/VFP5* transcripts in the analysis of the RNA-seq data because the purpose of this analysis was to uncover the effects of *VFP3/VFP5* suppression on plant transcriptome whereas suppression of *VFP3/VFP5* themselves does not represent the effect, but is the cause of the effect.

**Fig 9:**
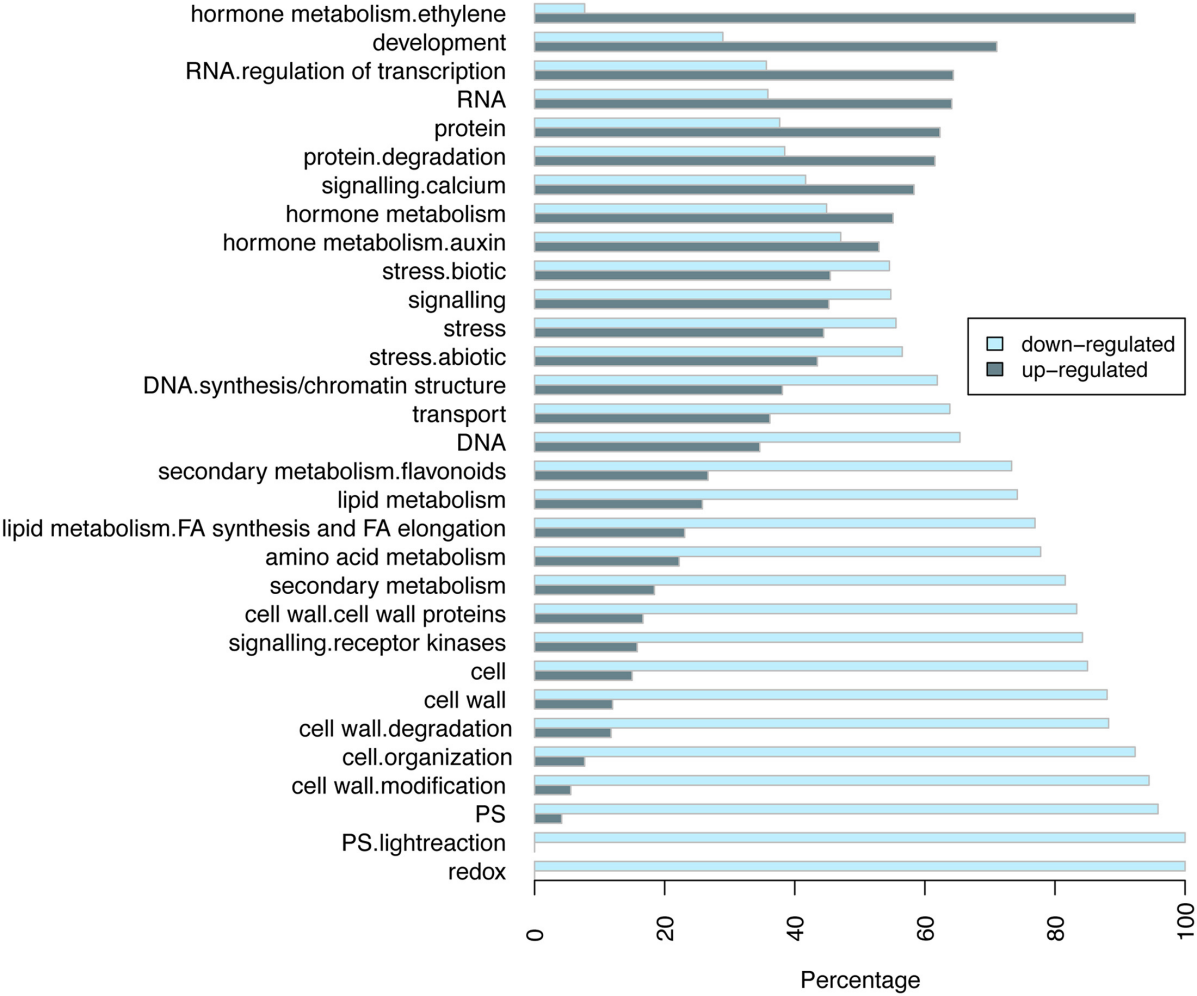
Percentage distribution of up- and down-regulated genes in *VFP3* RNAi-1 plants as compared to the wild-type Col-0 plants. Annotation is based on MapMan categories. Categories with gene number less than 10 are not shown. Gray bars indicate up-regulated gene categories, and blue bars indicate down-regulated gene categories.

**Fig 10:**
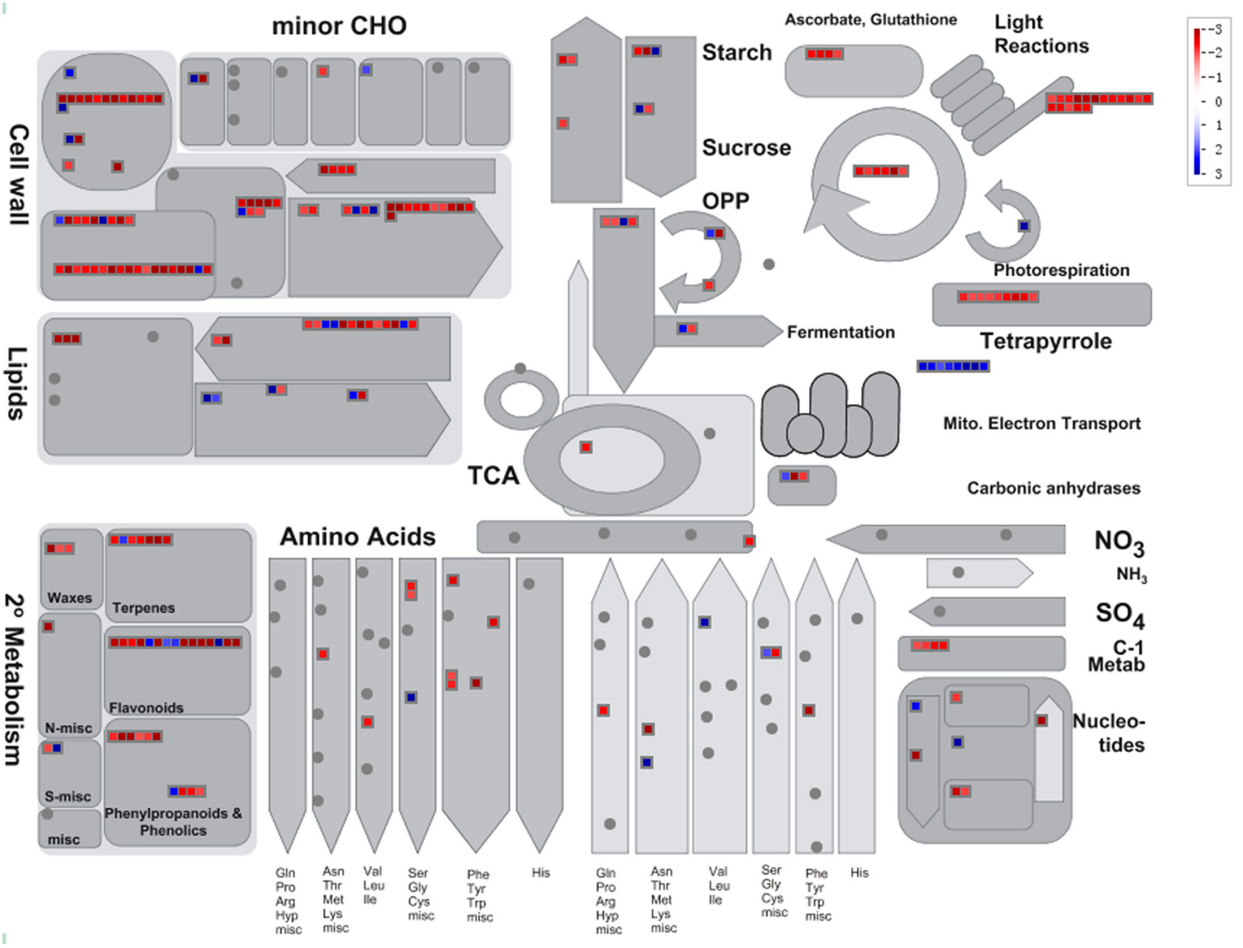
Metabolism overview of genes differentially expressed in *VFP3* RNAi-1 plants. The analysis employed the MapMan software. Values are log2 fold changes between the analyzed plants. Blue indicates up-regulation in gene expression, and red indicates down-regulation in gene expression.

We then used MapMan annotation (TAIR10) to assign genes to functional categories and performed function enrichment analysis on the differentially expressed genes. The 507 genes up-regulated in the *VFP3* RNAi-1 line were enriched for MapMan bins that included genes implicated in development, hormone metabolism (e.g., auxin and ethylene), RNA regulation of transcription, as well as genes associated with calcium transport (Table 1, S1 Table, and Fig 9). The 611 genes down-regulated in *VFP3* RNAi-1 leaves were enriched in MapMan bins that included genes related to amino acid metabolism, Calvin cycle and light reaction of photosynthesis, tetrapyrrole synthesis, and enzymes involved in redox, cell wall, secondary metabolism and lipid metabolism, as well as hormone metabolism (e.g., cytokinin and jasmonate) and metabolite transporters (Fig 9 and Table 1, S2 Table). One common trend within this large number of highly diverse genes is that many of them affect light signaling, calcium signaling, secondary metabolism, and/or redox state (Figs 9 and 10), all of which are often associated with states of stress [25, 39, 40]. This suggests that *VFP3* RNAi-1 plants are stressed and that the *VFP3/VFP5* genes are involved in regulating the plant cell homeostasis.

**Table 1:** Summary of functional categories of 507 up-regulated and 611 down-regulated DEGs in VFP3 RNAi plants. Numbers represent -log10(q-value).

Functional category	DEGs_up_regulated	DEGs_down_regulated
amino acid metabolism	0.00	2.84
amino acid metabolism.degradation	0.00	1.52
amino acid metabolism.synthesis	0.00	1.64
cell wall	0.00	31.71
cell wall.cellulose synthesis	0.00	3.46
cell wall.cell wall proteins	0.00	8.40
cell wall.degradation	0.00	5.76
cell wall.modification	0.00	12.10
cell wall.pectin*esterases	0.00	2.10
development	2.57	0.00
hormone metabolism	5.60	2.69
hormone metabolism.auxin	1.50	0.00
hormone metabolism.cytokinin	0.00	1.36
hormone metabolism.ethylene	5.60	0.00
hormone metabolism.jasmonate	0.00	1.62
lipid metabolism	0.00	4.41
lipid metabolism.FA synthesis and FA elongation	0.00	3.83
major CHO metabolism	0.00	1.46
PS	0.00	10.27
PS.calvin cycle	0.00	3.97
PS.lightreaction	0.00	7.29
redox	0.00	3.52
RNA.regulation of transcription	1.75	0.00
secondary metabolism	0.00	9.30
secondary metabolism.flavonoids	0.00	4.79
secondary metabolism.phenylpropanoids	0.00	1.59
secondary metabolism.simple phenols	0.00	1.67
secondary metabolism.wax	0.00	1.52
tetrapyrrole synthesis	0.00	5.52
transport	0.00	1.51
transport.calcium	1.31	0.00
transport.metabolite transporters at the envelopemembrane	0.00	1.78

## Discussion

Besides being an invaluable, and often the only, tool for genetic transformation of plants for agriculture and research, Agrobacterium-mediated genetic transformation represents a fascinating biological system for studies of a wide spectrum of basic processes in host cell biology, from nuclear import to proteasomal degradation to DNA repair [41]. The transformation process largely depends on the presence and activity of several bacterially-encoded effector proteins that are exported into the host cell together with the transforming T-DNA. One of such effectors is VirF, a virulence protein that contains the conserved F-box motif. In fact, VirF was the first prokaryotic F-box protein discovered [6], and it is presumed to facilitate proteasomal uncoating of associated bacterial and host proteins from the T-DNA [7, 8]. However, as Agrobacterium exports only a very limited complement of effector proteins into the host cell, and by analogy to many multifunctional effectors of other pathogenic bacteria [15–18], VirF may fulfill multiple roles in the infection process. We have addressed this potential functional diversity of VirF by systematically identifying its interactors in the host cell. This study reports one such identified interactor, VFP3, and its close homolog VFP5. VFP3 is a trihelix-domain transcription factor that binds to VirF most likely outside its F-box domain. VirF interacted with VFP3 and VFP5 inside living plant cells, in which these complexes accumulated in the nucleus. Importantly, the VirF-VFP3/VFP5 interaction did not activate the bona fide UPS as it did not destabilize VFP3 or VFP5. Whereas the exact effect of this interaction remains to be determined, the regulation of target protein activity by F-box proteins without proteolysis has been reported previously [42], and it cannot be ruled out that VirF functionality toward VFP3/VFP5 could be achieved in a similar manner. Alternatively, VirF, by virtue of its binding to VFP3, and likely to VFP5 as well, may alter the function of VFP3/VFP5 as transcriptional regulator(s). This, in turn, might affect at least some of the numerous VFP3/VFP5 gene targets, potentially making the plant more susceptible to infection. That we did not detect changes in Agrobacterium tumorigenicity following RNA silencing of *VFP3* expression suggests that other trihelix proteins, such as SIP1, can at least partly compensate for the loss of VFP3. For example, tumor growth can cause considerable stress to the host plant, and SIP1 interaction the Agrobacterium 6b oncogene [29, 30] may serve to relieve this stress and allow the host plant to tolerate the tumor better. Alternatively, the residual amounts of VFP3 were simply sufficient to support efficient infection.

Our RNA-seq data are significant beyond the potential role of VFP3/VFP5 in the interaction with VirF. While trihelix transcription factors initially were linked to the regulation of light-responsive genes, their range of functions has expanded to include stress responses and fine tuning of specialized developmental processes [25]. Our RNA-seq analysis of the *VFP3* RNAi-1 line examined the full range of involvement of trihelix proteins VFP3 and VFP5 in plant transcription. This is because the effect of reducing *VFP3* expression on the plant transcriptome likely also reflects at least partial reduction in the expression of *VFP5*. However, the contribution of VFP5 may be relatively minor because this gene is weakly expressed in leaves and it was only silenced 2-fold in the *VFP3* RNAi-1 line. Nevertheless, our data does show that VFP3/VFP5, directly and/or indirectly, affect the expression of over one thousand genes that have been implicated in a wide range of metabolic processes, including both up-regulation and down-regulation of genes with diverse functions in plant primary and secondary metabolism. The overall pattern of these differentially expressed genes is characteristic of cells experiencing stress. This suggests that VFP3 and/or VFP5 may be involved in maintaining cellular homeostasis and their deficiency results in general stress. Indeed, the lack of loss-of-function mutants of *VFP3/VFP5*, combined with our inability to recover RNAi plants with higher levels of suppression, suggest that plants which had lost the function of these proteins are unable to survive. This notion is lent further support by our data that even relatively modest levels of suppression affected expression of substantial numbers of genes, many of which were indicative of overall stress. Consistently, previous observations indicate that trihelix proteins participate in a wide spectrum of responses to biotic and abiotic stress, such as pathogen infection [25], cold- and salt-induced stress, osmotic stress, drought [25, 43–46], and hypoxia [47]. Thus, the gene expression changes observed in the *VFP3* RNAi-1 plants are likely mitigated by other trihelix family member(s) of the same SIP1 clade, or even other, more distant clades. Together, these data afford an insight into the potential range of functional complexity of the plant-specific family of trihelix DNA-binding domain transcription factors.

## Material and Methods

### Plant growth

Wild-type *Arabidopsis thaliana* (ecotype Col-0) and *Nicotiana benthamiana* plants were first grown on MS [48] aseptic medium and, after two weeks, transferred to soil and maintained in an environment-controlled chamber at 22°C–24°C under standard conditions of 16 h light (70–80 μmol photons m^-2^ s^-1^) and 8 h dark.

### Yeast two-hybrid system

To produce mutVirF, carrying within its F-box domain amino acid substitution mutations L26A and P27A, the corresponding fragment of the VirF coding sequence first was amplified using forward primer 5’ACGGGTCGACATGAGAAATTCGAGTTTGCGTG3’ and reverse primer containing the mutations 5’CAGCACGTGGTCTGCCGCATTTAGTAATTCTG3’. The resulting fragment was then used as a forward megaprimer, together with the reverse primer 5’ATATGGATCCTCATAGACCGCGCGTTGATCGA3’, to amplify the full mutVirF sequence, which was cloned into the SalI-BamHI sites of pSAT6-MYC-C1 [32]. To generate mutVirFdel1, lacking the 15 N-terminal amino acid residues, the mutVirF coding sequence was amplified using the primer pair 5’GCCGGAATTCCAGGTTCCCCACAAAACAGAAT3’/5’ATATGGATCCTCATAGACCGCGCGTTGATCGA3’ and subcloned back into the SalI-BamHI sites of pSAT6-MYC-C1. Finally, for LexA fusions, the mutVirFdel1 and VirFdel1 coding sequences were amplified and cloned into the EcoRI-BamHI sites of pSTT91 (TRP1+) [49]. For LexA-VFP3 fusion, the *VFP3* cDNA was amplified and cloned into the EcoRI-BamHI sites of pGAD424 (LEU2+, Clontech; Mountain View, CA). Constructs expressing Gal4AD-TMV MP and LexA-AtCUL1 fusions were described previously [32].

For yeast two-hybrid experiments, the potential interactors were introduced into the *Saccharomyces cerevisiae* strain TAT7 (L40-ura3) [20] and grown for 2 days at 30°C on a leucine-, tryptophan- and histidine-deficient medium in the presence of 6 mM of 3-amino-1’, 2’, 4’ triazole (3-AT). Positive interactions were detected by histidine prototrophy [50]. For identification of VirF interactors, a cDNA library from Arabidopsis Col-0 in pGAD424 [21] was screened with LexA-mutVirFdel1 as a bait as described [11, 20, 21].

### Agroinfiltration and microbombardment

For agroinfiltration, *Agrobacterium* EHA105 strain harboring the tested expression construct(s) was grown in LB medium supplemented with spectinomycin (100 *μ*g/ml) overnight at 28°C. Cells were harvested by centrifugation and resuspended to optical density of A_600_ = 0.1 in infiltration buffer [10 mM MgCl_2_, 10 mM MES (pH 5.5), 100 μM acetosyringone]. Bacterial suspension was incubated for 2 h at room temperature and infiltrated into the abaxial sides of 3- to 4-week-old intact *N*. *benthamiana* leaves with a 1-ml needleless syringe. Plants were grown for 48–72 h under standard growth conditions before being harvested.

For biolistic delivery, DNA preparations of the tested constructs were mixed at a 1:1 w/w ratio, and 100 *μ*g DNA was adsorbed onto 10 mg of 1-μm gold particles (Bio-Rad, Hercules, CA). These microprojectiles were bombarded into the leaf epidermis of *N*. *benthamiana* using a portable Helios gene gun system (Model PDS-1000/He, Bio-Rad) at a pressure of 90–150 psi, and tissues were analyzed 48 h after microbombardment.

### BiFC and subcellular localization

For BIFC, the coding sequences of VFP3 and At3g58630 were amplified using primer pairs 5’ATATGAATTCATGGAGACGACGCCGGAGAC3’/5’ATGCGGATCCTTACCTGAAGCAGCTCTT3’ and 5’AGCGGAATTCTATGGACACCGTCAACGATTC3’/5’GCGCGGATCCCTAGAAGTAACTAGGGAAAT3’, respectively, and the coding sequence of VFP5 was amplified using primer pair 5’ATATGAGCTCAAATGGAGACGACGACGCCGCA3’/5’ATGCGGTACCCTAGACTTTTCCTTGCCAGA3’, and cloned into the EcoRI-BamHI and SacI-KpnI sites, respectively, of pSAT6-nEYFP-C1 [23]. The coding sequences of VirF and 6b were amplified using primer pairs 5’ACGGGTCGACATGAGAAATTCGAGTTTGCGTG3’/5’ATATGGATCCTCATAGACCGCGCGTTGATCGA3’ and 5’ATGCGAGCTCAAATGACGGTAGCTAATTGGCAGG3’/5’ATGCGGTACCTTATGCGGAAAGATCGCATGAC3’, respectively, and cloned into the SalI-BamHI and SacI-KpnI sites, respectively, of pSAT6-cEYFP-C1 [23]. The tested combinations of these constructs were transiently expressed in *N*. *benthamiana* leaves by microbombardment.

To analyze the subcellular localization of VFP3, its coding sequence was amplified with the primer pair 5’ATATGAATTCATGGAGACGACGCCGGAGAC3’/5’ATGCGGATCCTTACCTGAAGCAGCTCTT3’ and cloned into the EcoRI-BamHI sites of pSAT5-ECFP-C1, which is identical to pSAT6-ECFP-C1 [51], except that its expression cassette is flanked by the I-CeuI sites. The resulting expression cassette was excised with I-CeuI and inserted into the pPZP-RCS1 binary vector [51, 52]. For transient expression of free DsRed2, an AgeI-KpnI fragment of pSAT6-DsRed2-C1 [51] was subcloned into the same sites of pSAT5A-masP-MCS-masT, which is identical to pSAT3A.masP.MCS.masT [53], except that its expression cassette is flanked by the I-CeuI sites. These constructs were transiently coexpressed in *N*. *benthamiana* leaves by agroinfiltration. BiFC signal and CFP and DsRed2 fluorescence were detected using a Zeiss LSM 5 Pascal confocal microscope. All experiments were repeated at least three times.

### Protein destabilization in a cell-free system

The VFP3 and VirF coding sequences were cloned into the EcoRI-BamHI sites of pSAT5-ECFP-C1 or SalI-BamHI sites of pSAT6-MYC-C1 [32], respectively. These expression cassettes were excised with I-CeuI or PI-PspI, respectively, and inserted separately or together into the binary pPZP-RCS1 vector [51]. These resulting constructs were transiently expressed for 72 h in *N*. *benthamiana* leaves by agroinfiltration, the leaves were then harvested and extracted, and cell-free degradation assay and western blot analysis were performed as described [31], using anti-GFP antibody (Clontech) followed by detection with secondary antibody conjugated to horseradish peroxidase (HRP). For loading controls, we used a major band at about 50 kDa, presumably representing the large chain of RuBisCO (ribulose-1,5-bisphosphate carboxylase oxygenase), detected on Coomassie blue-stained gels. Protein amounts were estimated by scanning densitometry of the corresponding western blot bands using the ImageJ software (version 1.49, NIH).

### Generation of *VFP3* RNAi plants

A 400-bp fragment between nucleotides 341 to 740 of the *VFP3* cDNA was amplified in two different variations, as an NcoI-AscI fragment and as a XbaI-BamHI fragment. Both fragments were inserted into the binary vector pFGC5941 (stock CD3-447, obtained from ABRC) in a forward and reverse orientation, respectively. The resulting construct was used to generate transgenic *A*. *thaliana* (ecotype Col-0) plants using the floral dip method [54]. Independent T1 transformants were selected on 1/2 MS medium, supplemented with BASTA (50 μg/ml^-1^) and transferred to soil. Their BASTA-resistant T2 progeny were verified for the presence of the transgene using primer pair 5’AGATGTTTCCCAGCGAGCTA3’/5’AGCATGCAAAAACCCTCAAT3’ and utilized for further analyses.

### Semi-quantitative RT-PCR

Leaves and roots were harvested from the wild-type Col-0 and *VFP3* RNAi-1 plants. Total RNA was extracted from these tissue samples using Trizol reagent (Invitrogen) and purified using the SV Total RNA Isolation System (Promega). The quality and quantity of the purified RNA was assessed using Biospec-Nano (Shimadzu, Kyoto, Japan), the preparations were aliquoted and stored at -80°C for future use. The reverse transcription (RT) reactions were carried out with 500 ng of the total RNA and the RevertAid RT kit (Thermo Scientific). The resulting cDNA was amplified for the indicated number cycles using the primer pairs 5’CGGAGACTCAGTCGAAGACTCA3’/5’CCAACCATTGCTCCTTGCTTCAC3’ specific for the *VFP3* gene, 5’GTATGGAGACGACGACGCCGCA3’/5’CTAGACTTTTCCTTGCCAGA3’ specific for the *VFP5* gene, or 5’AGAGATTCAGATGCCCAGAAGTCTTGTTCC3’/5’ AACGATTCCTGGACCTGCCTCATCATACTC3’ specific for *ACTIN2* as an internal control of a constitutively expressed gene.

For detection of *VFP3* and *VFP5* transcripts in the leaf tissues, we used the following PCR conditions: 1 cycle at 94°C for 3 minutes, 1 cycle at 94°C for 30 seconds, 1 cycle at 55°C for 30 seconds, 1 cycle at 55°C for 30 seconds, the indicated number cycles (i.e., 20, 25, 30, or 35) at 72°C for 1 minute, and 1 cycle at 72°C for 5 minutes. For detection of *VFP3* transcripts in the root tissues, the PCR conditions comprised 1 cycle at 95°C for 2 minutes, 6 touch-down cycles at 94°C for 45 seconds, 62°C for 45 seconds (this temperature is reduced by 1°C per touch-down cycle), and 72°C for 5 minutes, the indicated number cycles (i.e., 20, 25, or 30) at 94°C for 45 seconds, 55°C for 45 seconds, and 72°C for 5 minutes, and 1 cycle at 72°C for 10 minutes. In addition to the *ACTIN2* control, each set of reactions included a no-sample negative control. The PCR products were resolved on agarose gels and quantified by scanning densitometry of the corresponding bands using the ImageJ software. The calculated amounts of the *VFP3* and *VFP5* transcripts were normalized to the amounts of *ACTIN2* transcripts.

### Tumorigenesis

Root segments from aseptically grown 15-20-day-old wild-type Col-0 and *VFP3* RNAi-1 plants (50–70 segments per plant) were submerged in liquid cultures of the indicated cell densities (i.e., A_600_ = 0.1, 0.01, and 0.001) of Agrobacterium strain LBA1010, incubated for 10 min at 25°C, cultivated for 48 h at 25°C in hormone-free MS (HFMS) medium, washed, cultured for additional 4 weeks in HFMS supplemented with 100 μg/ml timentin, and scored for tumors. Each experiment was repeated three times.

### High-throughput cDNA sequencing (RNA-seq)

Total RNA from the leaves of the wild-type Col-0 and *VFP3* RNAi-1 plants was extracted using Trizol reagent (Invitrogen) and purified using the SV Total RNA Isolation System (Promega). Polyadenylated RNA was isolated from the purified total RNA using two rounds of purification with oligo-dT attached to magnetic beads. During the second elution, the purified RNA is also fragmented and primed for cDNA synthesis. This RNA preparation (1.2 ng) was used for RNA-seq library construction according the manufacturer’s recommendations (Illumina). Briefly, random hexamer primers were used to reverse-synthesize the first strand of cDNA, followed by the second strand synthesis, and double-stranded cDNA was separated from the reaction mix using AMPure XP beads (Beckman Coulter). After ligation of adaptors, selective PCR was performed to enrich for the DNA fragments that have adapter molecules ligated to both ends as well as to amplify the amount of DNA in the library. cDNA fragments of approximately 200–500 bp were isolated by gel electrophoresis, amplified by 15 cycles of PCR and PCR Primer Cocktail (Illumina), and sequenced on the Illumina NextSeq500 platform. Three biological replicates were used for all RNA-seq experiments.

### Read mapping and data analysis

Adapters were removed from raw sequence reads using FASTX-toolkit pipeline version 0.0.13 (http://hannonlab.cshl.edu/fastx_toolkit/). Sequence quality was examined using FastQC (http://www.bioinformatics.babraham.ac.uk/projects/fastqc/), and low quality reads were filtered also using FASTX-toolkit setting parameters as “q20p80”, i.e., for each retained read, 80% of bases must have sequence quality greater than 20, which corresponds to 1% sequencing error rate. These reads were then mapped to the Arabidopsis genome (TAIR10.22), obtained from EnsemblPlants (http://plants.ensembl.org) using TopHat version 2.0.10 (http://tophat.cbcb.umd.edu/) [55]. Raw count data were obtained by Cuffdiff embed in Cufflinks pipeline version 2.1.1 (http://cufflinks.cbcb.umd.edu) [56]. Differentially expressed genes (DEGs) were identified by DESeq [38] using Bioconductor (http://www.bioconductor.org), based on comparison between the wild-type and VFP3 RNAi plants, and setting the false discovery rate (FDR) less than 0.001 and absolute value of log_2_FC (fold-change) greater than 2.

Arabidopsis loci were then functionally annotated and classified into hierarchical categories using the MapMan functional classification system [57]. For each category, we assigned DEGs into two groups of up- and down-regulated genes, calculated their percentages, and plotted their distributions. Categories with gene number less than 10 were not included in the presented data. Over-represented functional categories enrichments were also conducted based on Fisher’s exact test as described [58]. Metabolism overview of DEGs was visualized using MapMan version 3.5.1 [57].

## Supporting Information

S1 Table Genes up-regulated in VFP3 RNAi-1 plants. (XLSX)

S2 Table Genes down-regulated in *VFP3* RNAi-1 plants. (XLSX)
